# Mycobacterial Dormancy Systems and Host Responses in Tuberculosis

**DOI:** 10.3389/fimmu.2017.00084

**Published:** 2017-02-15

**Authors:** Vidyullatha Peddireddy, Sankara Narayana Doddam, Niyaz Ahmed

**Affiliations:** ^1^Pathogen Biology Laboratory, Department of Biotechnology and Bioinformatics, University of Hyderabad, Hyderabad, India; ^2^Laboratory Sciences and Services Division, International Centre for Diarrhoeal Disease Research Bangladesh (icddr,b), Dhaka, Bangladesh

**Keywords:** *Mycobacterium tuberculosis*, dormancy, DosR regulon, granuloma, antibiotic resistance, alveolar macrophages, latency

## Abstract

Tuberculosis (TB) caused by the intracellular pathogen, *Mycobacterium tuberculosis* (*Mtb*), claims more than 1.5 million lives worldwide annually. Despite promulgation of multipronged strategies to prevent and control TB, there is no significant downfall occurring in the number of new cases, and adding to this is the relapse of the disease due to the emergence of antibiotic resistance and the ability of *Mtb* to remain dormant after primary infection. The pathology of *Mtb* is complex and largely attributed to immune-evading strategies that this pathogen adopts to establish primary infection, its persistence in the host, and reactivation of pathogenicity under favorable conditions. In this review, we present various biochemical, immunological, and genetic strategies unleashed by *Mtb* inside the host for its survival. The bacterium enables itself to establish a niche by evading immune recognition *via* resorting to masking, establishment of dormancy by manipulating immune receptor responses, altering innate immune cell fate, enhancing granuloma formation, and developing antibiotic tolerance. Besides these, the regulatory entities, such as DosR and its regulon, encompassing various putative effector proteins play a vital role in maintaining the dormant nature of this pathogen. Further, reactivation of *Mtb* allows relapse of the disease and is favored by the genes of the Rtf family and the conditions that suppress the immune system of the host. Identification of target genes and characterizing the function of their respective antigens involved in primary infection, dormancy, and reactivation would likely provide vital clues to design novel drugs and/or vaccines for the control of dormant TB.

## Introduction

Tuberculosis (TB), a chronic infectious disease caused by *Mycobacterium tuberculosis* (*Mtb*), is one of the major drivers of human mortality worldwide since many decades with an estimated global burden of 10.4 million new TB cases and 1.4 million TB deaths in the year 2015 (1). Due to the growing efficiency of case finding and in the aftermath of DOTS regimen, the mortality rate decreased worldwide by 22% during 2000–2015 (1). There has been a decrease in the prevalence of TB cases dramatically from 4 million to 2.8 million cases in the last decade with a decrease in TB mortality form 330,000 cases to 220,000 cases, annually, in India (2). However, the burden of disease in the form of active TB still persists at an alarming rate in low and middle income countries with an estimated 580,000 new cases due to multiple drug resistant TB (MDR-TB), globally (1). TB infection is caused by the inhalation of aerosolized particles harboring *Mtb*. Various factors such as host’s immune status, inhaled bacillary load, the closeness of contact, and infectiousness of the source case play a primary role in TB transmission (3). The ability of inhaled *Mtb* aerosolized particles to infect the phagocytic immune cells [dendritic cells (DCs) and macrophages] and the non-phagocytic alveolar endothelial cells such as M cells and type 1 and type 2 epithelial cells (pneumocytes) (4) allows *Mtb* to replicate within the macrophages and spread to pulmonary lymph nodes and to several extra pulmonary sites before the adaptive immunity sets in (5). Hence, multiple possibilities exist where there could be (a) bacterial clearance by host immune activation, (b) multiplication of bacteria leading to primary infection, (c) dormant survival of bacteria rendering the host non-contagious and asymptomatic, and (d) reactivation of bacteria by infringement of dormancy causing re-emergence of the infection (6). Dormancy of the *Mtb* in the host is largely attributed to its sophisticated immune-evading capability that allows it to persist indefinitely. The key strategies adopted by *Mtb* to maintain its dormant phase include manifestation of immune [manipulation of toll-like receptor (TLR), cytokine, and immune cell function], biochemical (development of resistance to reactive intermediates and antibiotics), and genetic (activation of dormancy-associated genes) mechanisms. Despite vaccination with BCG (which is effective only in children) and the availability of powerful drugs to treat *Mtb*, there has been no decrease in the global burden. Epidemiological studies indicate that 90–95% of new *Mtb* infections could become dormant, and this dictates the immunological poise between the pathogen and the host (7). Besides persistent infections due to evolution of multidrug resistant and extensive drug resistant *Mtb*, a large reservoir of population hosting *Mtb* in the dormant stage represent the prime cause of new TB cases throughout the world (8). Hence, diagnosis and treatment of individuals hosting *Mtb* in a dormant stage is one of the crucial strategies to be adopted for the prevention of TB. Diagnostic methods such as tuberculin skin test (TST) and cell-mediated immune response-dependent approaches were developed based on the current understanding of the mechanisms that contribute to the establishment of persistent infection (9). The latest developments in understanding the cellular, biochemical, and molecular mechanisms that are employed for the establishment of dormant stage by *Mtb* are discussed in this review.

## Immunology of Dormant *Mtb*

### Evading Immune Detection

#### Masking: An Immunological Disguise

Mycobacteria adopt multiple strategies to avoid the attack from macrophages. They express surface lipids such as phthiocerol dimycoceroserate, which can mask the pathogen-associated molecular patterns (PAMPs), thereby going “unnoticed” by the innate immune system (10). The phenolic glycolipid produced by these bacilli induces the production of the chemokine CCL2 to recruit macrophages for further infection (10). In the upper airway where a constant and heavy recruitment of macrophages occurs due to the presence of TLR stimulating bacteria, thus posing a very hostile environment, *Mtb* adopts a different immune evasion strategy by forming small infection droplets that allow them to be delivered directly into the alveolar spaces of the lower lung, which anchorages a few microbicidal macrophages (11).

### Manipulating the TLR Responses

In the macrophages, which are the crucial niche for replication, *Mtb* interacts with various receptors to initiate phagocytosis. Despite the bactericidal properties of the macrophages, *Mtb* employs phagocytosis as a primary mode of gaining entry to establish the niche. The opsonization of the bacillus by the complement or antibodies determines the nature of receptors engaged and also the nature of events that are involved in the outcome of the infection. Recognition of *Mtb* through its cell wall glycolipids involves the formation of TLR heterodimers (12). The importance of TLR-mediated signaling during *Mtb* infection is well proven in various TLR knockout animal models (13). Mycobacterial components such as lipomannan, lipoarabinomannan (LAM), 38- and 19-kDa mycobacterial glycoproteins, and phosphatidylinositol mannoside (PIM) induce the formation of TLR1/6 heterodimer (12). The 38- and 19-kDa mycobacterial glycoproteins, PIM, and triacylated lipoproteins favor the formation of TLR2/TLR1, whereas the diacylated lipoprotein induces TLR2/TLR6 dimerization (13). The susceptibility to *Mtb* infection is also due to genetic polymorphisms in the host genes (14). It is well established that *Mtb* has the ability to modulate the immune responses to its advantage. Exposure of THP-1 cells to *Mtb* cell wall components results in the *de novo* synthesis of TLR4, thereby decreasing the production of Th1 cytokines (15). Induction of apoptosis in bystander cells during *Mtb* infection of macrophages is a classic example of how this pathogen causes immunosuppression in infected individuals, thereby gaining the survival advantage (16). Interaction of *Mtb* cell wall components with TLRs modulates a number of events that include antigen presentation (17), phagolysosomal fusion (13), apoptosis of macrophages (12), and production of reactive oxygen and nitrogen intermediates (18).

Although Myd88-dependent signaling of TLRs is well established in mycobacterial pathogenesis, recent studies indicate independent roles for Mal (the TLR adaptor) and Myd88. Individuals with the single-nucleotide polymorphisms, D96N and S180L, in the TIRAP gene (that codes for Mal) display differential susceptibility to *Mtb*. Heterozygous genotype was associated with a protection toward TB, whereas the homozygous genotype was related to susceptibility (19). Using a murine model that carried the human equivalent mutation in TIRAP gene, it was demonstrated that in the homozygous genotype for the mutation, the mycobacterial load was higher and this was independent of the macrophage cytokine production (20). Further, *in vitro* studies indicated that mutation in TIRAP gene affected phagosome maturation and intracellular killing of *Mtb* (20).

#### Antigen Presentation by MHC

The TLR2-dependent surface expression of MHC class II receptor and their antigen-presenting ability was found to be inhibited by either *Mtb* infected or 19-kDa lipoprotein (LpqH) exposed macrophages (12, 21). Class II transactivator (CIITA), a TLR-2-dependent regulator of MHC class II a, b, invariant chains contributes to antigen processing and its expression was found to be decreased during *Mtb* infection (22–24). Its importance is further strengthened with the observation that CIITA knockout mice could not survive *Mtb* infection (25). *Mtb* also inhibits the expression of genes involved in MHC class II processing and presentation (26, 27) and the posttranslational function of these molecules. Another interesting feature by which *Mtb* evades TLR-mediated immune response is by the differential antigen-presenting ability of MHC class II. It is demonstrated in *Mtb*-infected lung DCs of mice that despite normal levels of MHC class II molecules, antigen presentation capability was decreased, whereas in macrophages, both the MHC class II molecules expression and antigen presentation capacity were found to be decreased (28–31).

#### Phagolysosomal Fusion/Fission

**Figure 1:**
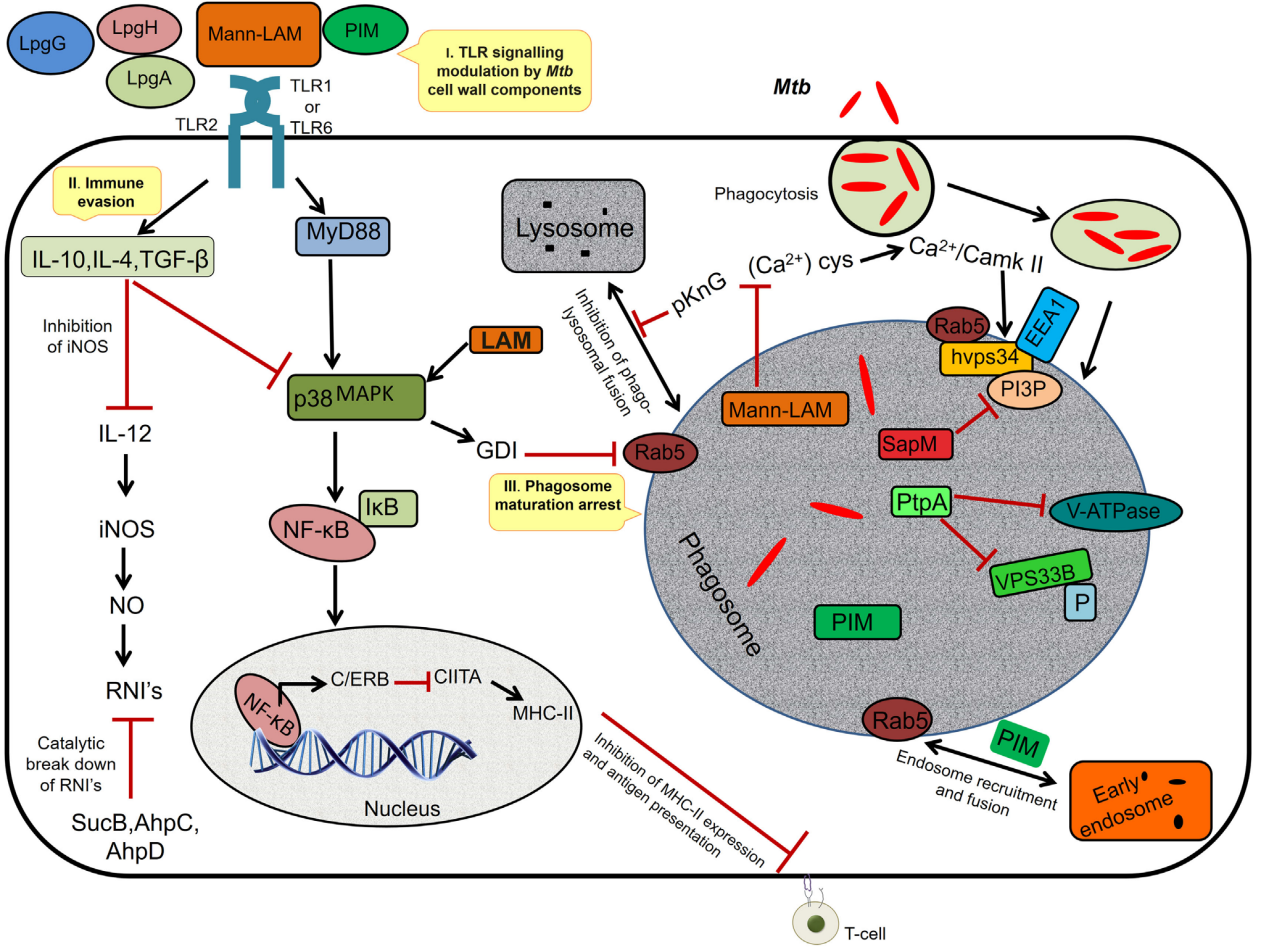
**I. *TLR signaling modulation***. The *Mycobacterium tuberculosis* (*Mtb*) cell wall components interact with TLR-2 and modulate the host cell signaling *via* p38 MAPK, resulting in activation of NF-κB and synthesis of C/ERB that binds to class II transactivator (CIITA) promoter and inhibits CIITA production leading to decreased expression of MHC-II, thus inhibiting antigen presentation. II. *Immune evasion*: prolonged signaling by cell wall components induces the anti-inflammatory cytokines, TGF-β, interleukin (IL)-10, and IL-4 (Th2-dependent manner), which inhibits IL-12. IL-12 is required for the production of interferon-γ, iNOS, and NO, a major defense of the host against *Mtb*. The intracellular pathogen secretes SucB, AhpC, and AhpD, which catalyzes the breakdown of reactive nitric intermediates (RNIs). III. *Phagosome maturation inhibition*: mannosylated-LAM (ManLAM) activates GDI *via* p38MAPK leading to the inhibition of Rab5 activity that is required for the recruitment of early endosomal autoantigen1 (EEA1); it also inhibits the increase in cytosolic Ca^2+^ flux required for the hvps34 activity. pknG prevents phagolysosomal fusion and sapM, ptpA inactivates the phosphatidylinositol 3-phosphate (PI3P) and VPS33B through dephosphorylation. Phosphatidylinositol mannoside (PIM) mediates the early endosomal fusion through which bacilli gains access to nutrients such as iron required for its survival.

During chronic infections, repeated stimulation of TLRs by the *Mtb* components such as mannosylated-LAM (ManLAM) and PIM causes phagosomal maturation and arrest allowing persistence of mycobacteria inside the phagosome (32) (Figure 1). Among the successful strategies adopted by *Mtb* to establish a niche in the host, inhibition of macrophage maturation is best characterized. The mycobacterial products (ManLAM, trehalose dimycolate, and sulfolipids), phosphatase SapM, kinase PknG, and early secretory antigenic target-6 (ESAT-6) have been implicated in the inhibition of macrophage maturation (33, 34). ManLAM inhibits Ca^+^ surge that modulates the calmodulin- and Ca^2+^/calmodulin-dependent kinase II-dependent delivery of early endosomal autoantigen1 (35–37), which in turn is necessary for the delivery of lysosomal hydrolases and vacuolar H^+^-ATPases into phagosomes. ManLAM blocks ESAT-6 recruitment by inhibiting PI3K hVPS34 to block PIP3 production as well as SapM-mediated dephosphorylation of phosphatidylinositol 3-phosphate (37–39). Further, phosphorylation of unknown substrates by the kinase PknG and activation of p38MAPK by LAM to reduce the levels of Rab5 are some other mechanisms by which *Mtb* inhibits macrophage maturation, though the exact role of ManLAM in these two mechanisms is not clear (40–42). Further, *Mtb* also disrupts the scaffolding of endosomes required for phagosome–endosome interactions leading to delay in phagosomal maturation (43, 44). Because of the abovementioned mechanisms, the phagosomal compartment formed in the macrophage is devoid of acidification and lysosomal enzymes, thereby allowing *Mtb* to successfully establish a niche for its growth and replication.

#### Apoptosis of Immune Cells and Redefining the Immune Cell Fate: A Process of Exploitation

Mycobacterial infection leads to various cellular fates such as apoptosis, necroptosis (type of programed necrosis), and autophagy (45–47). In the macrophages, apoptosis and autophagy are the natural defense mechanisms that operate to eliminate microbial infection and invasion. The spread of these bacilli is lowered (46) by tumor necrosis factor (TNF)-α activated caspase 8-mediated extrinsic cell death pathway that involves kinases p38, ASK1, and c-Abl (48). Additionally, autophagy also promotes the clearance of *Mtb* (47, 49), which is supported by the observation that the survival of *Mtb* depends on the expression pattern of factors predominantly involved in autophagy (50). However, mycobacterial species have evolved mechanisms that prevent apoptosis and the autophagy of immune cells, so that they can survive in these cells and remain dormant for longer durations (51). Interestingly, the extent of alveolar macrophage apoptosis and *Mtb* virulence are inversely correlated. *Mtb* H37Rv, a virulent strain, inhibits apoptosis by enhancing the release of membrane-bound TNFR2 receptors (52, 53) and also by upregulating the expression of Mcl-1 protein, a member of antiapoptotic B-cell lymphoma/leukemia 2 family (54). The products encoded by certain *Mtb* genes also influence the apoptosis of infected macrophages. Overexpression of the *Mtb* type I NADH dehydrogenase (nuoG) neutralizes NOX2-derived reactive oxygen species (ROS) (55, 56) and thereby inhibits apoptosis in macrophages. Such a role was also observed in PknE and SecA2 genes using knockout strategies (57, 58).

Apoptosis is induced in host cells in response to pathogen infection, which determines the initiation of infection, survival, and escape from the host. An interesting feature during this process is the induction of apoptosis in uninfected cells due to the non-specific activation of cytokines (59). Thus, the induction of apoptosis of T cells (bystander cells) by *Mtb* is beneficial for its survival (16). In T cells stimulated with non-specific phytohemagglutinin (PHA) or specific culture filtrate protein of *Mtb*, apoptosis was evident in non-specifically stimulated T-cells that were dependent on Fas–Fas ligand interactions. Further, a significant release of TNF-α indicated its association with specific T-cell apoptosis during *Mtb* infection of macrophages (16).

*Mtb* exerts altogether a different kind of action on T-cells (CD4^+^ and CD8^+^) to delay T-cell responses by inhibiting apoptosis. Evidence for the ability of *Mtb* to inhibit apoptosis is indicated by (a) the promotion of development of CD8^+^ T cell responses by the Sec2A-deficient mutant strain of *Mtb*; (b) manipulation of eicosanoid metabolism of T cells (60); (c) increased frequency of macrophage apoptosis, accelerated CD4^+^ and CD8^+^ T-cell responses, and enhanced control of bacterial burden in Alox5 (5-lipoxygenase, required for generation of LXA4) deficient mice infected with virulent *Mtb* (61); and (d) enhanced susceptibility to *Mtb* infection due to polymorphisms in Alox5 and lta4h (62, 63). Another important mechanism by which *Mtb* enhances its survival in the host is to delay the expansion of Foxp3^+^ regulatory T (Treg) cells and thereby delay adaptive immunity (64). Although inflammation and cytokines produced during *Mtb* infection play a role in Treg cell proliferation, Treg cells that recognize *Mtb* antigens expand preferentially. It is reported that mice infected with wild-type *Mtb* displayed higher Treg cell proliferation than those infected with a virulent strain of *Mtb* deficient in expression of the specific antigen, Ag85B (64). Further, increased bacterial load associated with delay in priming of effector T cells was observed in mice receiving *Mtb*-specific Treg cells and such an effect was not observed in mice that received *Mtb*-specific Foxp^3−^ CD4^+^ T cells (64). These observations clearly indicate that *Mtb* expresses certain antigens and allows Treg cells specific to these antigens to proliferate rapidly and limit the rate of effector T-cell priming and expansion at this site.

Migration of DCs from the lungs to the lymph node precedes CD4^+^ T-cell responses during *Mtb* infection, and this was demonstrated in interleukin (IL)-12p40-deficient mice (65). Further, the restoration of the ability of DCs and activation of CD4^+^ T cells upon treatment with IL-12p40 (65) and presence of the same subset of DCs in the lymph nodes that were primed in the lungs (30) provides further evidence for the acquisition of bacilli and trafficking by DCs as a rate limiting step in the initiation of adaptive immunity. An inflammatory stimulus that resulted in migration of DCs from the lung to the lymph node failed to accelerate the delivery of *Mtb* from lung to lymph nodes, suggesting that DCs infected with *Mtb* are intrinsically impaired to migrate (65).

#### Manipulation of Host Cytokine Responses

*Mtb* infection manipulates host cytokine responses in different directions to create a balance and take advantage for its survival. Prolonged signaling of TLR2 by *Mtb* cell wall components results in increased production of IL-10, IL-4, and TGF-β, which then inhibits the IFN-γ-mediated activation of macrophages (27, 66–69), thereby the immune surveillance of T cells is evaded. Recognition of mycobacterial PAMPs by macrophages stimulates the production of cytokines like type I interferons (IFNs) and TNF (70, 71) through the TLR2 pathway, and these two cytokines promote apoptosis and necroptosis. Host cytokine responses are manipulated by various molecules during *Mtb* infection (72). It is demonstrated that IFN-γ and ESAT-6 inhibits TNF-α, IL-17 production, and early expression markers on T cells (73). Tim-3 inhibits the expansion of Th1 cells to prevent production of excess pro-inflammatory cytokines (74). Further, programed death-1 (PD-1) inhibits CTL function during Mtb infection (75). *Mtb* evolved mechanisms to control the expression of ESTAT-6, Tim-3, and PD-1 to control the manipulation of cytokine responses.

Increased production of TNF-α by macrophages induces generation of mitochondrial ROS, which confers the antimicrobial properties and necroptosis of these cells during *Mtb* infection (76–78). TNF-mediated ROS generation is brought through receptor-interacting protein 1 (RIP1), receptor-interacting protein 3 (RIP3), phosphoglycerate mutase family member 5, mixed lineage kinase domain-like protein (MLKL), and dynamin-related protein-1-dependent pathways (77). In brief, binding of TNF-α to its receptor results in the formation a membrane-proximal super-molecular structure complex 1 [containing TNF receptor-associated death domain (TRADD) that binds to RIP1, TNF receptor-associated factor 2/5 (TRAF2/5), and cellular inhibitor of apoptosis 1/2 (cIAP1/2)] followed by polyubiquitination of RIP1 or TRAF2 by cIAPs, which then allows NF-κB translocation into nucleus to initiate transcription of A20 and cylindromatosis (CYLD) (79, 80). Deubiquitination of RIP1 by A20 and CYLD (79) results in complex I getting converted to complex II [containing RIP1, Fas-associated protein with death domain (FADD), caspase-8, and TRADD] (80). Apoptosis is initiated by the activated caspase-8 of this complex and in situations wherein this activity is abrogated, RIP1 and RIP3 come together in complex III also with FADD, caspase-8, and TRADD to form a necrosome, in which RIP1 phosphorylates RIP3 and further engages MLKL, leading to necroptosis (80). *Mtb* targets the caspase-8 activity and leading thereby the macrophages to undergo necroptosis instead of apoptosis, since the former process is favorable to its survival (81). Inducing a very high level of TNF-α and promoting the secretion of a biological factor that can block caspase-8 activity have been proposed to be reasons for the ability of virulent *Mtb* to favor occurrence of necroptosis (82).

Other cytokines that are implicated in *Mtb* pathophysiology are IL-10 and IL-4/IL-13. IL-10, also referred to as “cytokine synthesis inhibitory factor,” produced by Th2 cells regulates macrophage and DC function in response to *Mtb* infection. The production of IL-10 during *Mtb* infection is more of an advantage to the pathogen than the host. IL-10 facilitates *Mtb* survival by inhibiting phagosome maturation thorough a STAT3-dependent and p38-independent mechanism (83), IFN-γ-mediated production of reactive oxygen and nitrogen intermediates (84), blocking antigen presentation by downregulating the expression of major histocompatibility complex molecules (85), DC migration (86), and recruitment of Th1 cells to the lungs by modulating CXCL10 production (87). In humans, IL-10 is responsible for limiting immune responses during *Mtb* infection (88). Further, an association between IL-10 gene polymorphism and susceptibility to TB was demonstrated (89). On the other hand, the *Mtb* strains HN878 and CH subvert the immune response *via* induction of IL-10 (90).

Besides the classical activation of macrophages modulated by many cytokines during *Mtb* infection, IL-4/IL-13 facilitates alternative activation of these cells. *Mtb* exploits the alternatively activated macrophages to divert the microbial actions of classically activated macrophages. Alternative activation results in induction of Arg1 gene whose protein product competes with iNOS for the substrate l-arginine (91). This results in lower production of NO reactive intermediates. Upregulation of IL-4/IL-13 was observed in patients with progressive pulmonary TB and in PBMCs infected with HN878 *Mtb* strain (92–94). IL-4/IL-13 induces Arg1 in alternatively activated macrophages to subvert the host NO-based mycobactericidal activity and could be a tactic by *Mtb* to thrive inside classically activated macrophages. On the other hand, the enhanced production of IL-4/IL-13 due to alternate activation of macrophages inhibits autophagy to facilitate the survival of *Mtb* (95).

#### Resistance to Reactive Nitrogen Intermediates

Generation of reactive nitrogen intermediates (RNIs) by the macrophages through nitric oxide synthase 2-dependent pathway mediated by IFN-γ is an antimicrobial strategy displayed by these cells and this process has been shown to be vital for the control of TB (96). In the macrophages, the inducible form of NOS is activated by the cytokines produced by Th1 lymphocytes that stimulate the production of nitric oxide (97), which reacts with superoxide radicals to form RNIs. The RNIs thus produced, attack bacterial macromolecules to aid in killing. Although the role of RNIs in the control of TB in humans is not yet clear, some studies provide substantial evidence that RNIs play a role in innate immunity mounted during mycobacterial infection. The susceptibility to TB was found to be associated with genetic alterations in the NOS2A gene (98). Further, the negative correlation of mycobacterial growth and NO production in human alveolar macrophages, elevated expression of NOS2 in the lungs of TB patients, and reactivation of dormant TB due NOS2 inhibition support the role of the RNIs mycobacterial pathogenesis (99). However, *Mtb* has developed mechanisms that can subvert the antimicrobial actions of the macrophages, which allows these bacilli to establish a niche and remain in the host for a long time. ManLAM was also described to trigger Th2 cytokines such as IL-4 and IL-10 that inhibit the action of inducible NO synthase, an enzyme critical for the production of NO (9).

The alkyl hydroperoxide reductase subunit C encoded by the mycobacterial gene AhpC, in association with peroxidase, peroxinitrite reductase, dihydrolipoamide dehydrogenase (Lpd), dihydrolipoamide succinyltransferase (SucB), and thioredoxin-like AhpD catalyzes the breakdown of RNIs to protect *Mtb* from the antimicrobial actions of the macrophages (100, 101). Further, the mycobaterial gene MsrA encodes methionine sulfoxide reductase, an enzyme that converts methionine sulfoxide [produced out of a reaction between peroxynitrite (ONOO−) and methionine residues of proteins] to methionine, protects bacteria against RNIs (102). *Rv1205*, a pupylated proteasome substrate, catalyzes the production of cytokinins and helps *Mtb* to defend against NO (103). To summarize, TLR agonists present in the thick cell wall of *Mtb* causes prolonged TLR signaling resulting in various immune evasion mechanisms. Among these mechanisms, inhibition of MHC class II appears to be predominant that allows preventing detection by CD4^+^ T cells.

### Antibiotic Tolerance: An Acquired Fitness Advantage

Besides the fixed genetic mutations that the *Mtb* undergo to develop antibiotic tolerance, another interesting feature adopted by *Mtb* to remain dormant in the host is the development of “phenotypic drug resistance” or simply called “drug resistance,” during which a transient resistance to antibiotics is developed (104, 105). This is achieved by induction of specific macrophage-induced efflux pumps (106). Adding to this, the same pumps promote intracellular bacterial growth, thereby providing a double advantage to the *Mtb*.

The role of toxin–antitoxin (TA) systems in conferring the “non-classical” antibiotic resistance to allow *Mtb* to remain in a non-replicating phase for longer durations is very interesting. This system encoded by two genes is composed of two proteins, namely, the long-lived protein “toxin” and the short-lived protein “antitoxin.” In *Mtb*, under normal physiological conditions, the toxin is neutralized by the antitoxin. To remain in dormant stage, *Mtb* represses the expression of the antitoxin protein resulting in the accumulation of toxin. Under such conditions, the toxin protein acts as a ribonuclease to cleave free and ribosomal bound single-stranded mRNA resulting in inhibition of protein synthesis and bacterial growth. This allows the *Mtb* to persist in the host without any signs of infection state for a long time. Multiple TA systems, approximately 88, exist in the bacilli and their numbers are ever increasing (107–109), with H37Rv strain found to harbor 38 modules of 5 TA systems (3 relBE, 24 vapBC, 8 mazEF, 1 higBA, and 2 parDE) (108). Over expression of these genes belonging to different modules in *Mycobacterium smegmatis* stopped the growth of the recipient cells to remain in latent phase. In general, the mycobacterial TA proteins are homologous to the proteins of other bacterial species and some of them exhibit certain special features. The MazF protein besides cleaving mRNA can also interact with DNA topoisomerase (110), thus indicating that differential mechanisms may exist in the way the TA proteins act to contribute to persistent infection in the host.

### Granuloma Formation: A Finely Negotiated Refuge?

Granulomas are formed in response to infectious and non-infectious stimuli and are associated with various diseases (111). They are aggregates of macrophages whose membranes are interlocked and their occurrence is more prevalent in TB worldwide. Although it was initially reported that granulomas are complex protective structures that contain host cells to wall off bacteria (104) and can also sterilize infection (112), many studies have indicated that they are conducive to heavy bacterial burdens in TB (113). In case of active disease, some of the lesions are cleansed by the host even though there is progression of other lesions indicating that lesional heterogeneity persists after the initiation of adaptive immunity where differential killing of the bacteria take place, deciding the outcome of the clinical infection (112). *Mtb* in fact enhances the formation of granulomas (114) for their expansion and dissemination. This is accomplished by spreading of *Mtb* from dying macrophages to newly recruited ones. Macrophages undergo apoptosis when the bacterial load reaches a threshold and the nascent granulomas are presented to the uninfected macrophages. Thus, the *Mtb* numbers are phenomenally expanded by way of macrophage death and re-phagocytosis (114).

**Figure 2:**
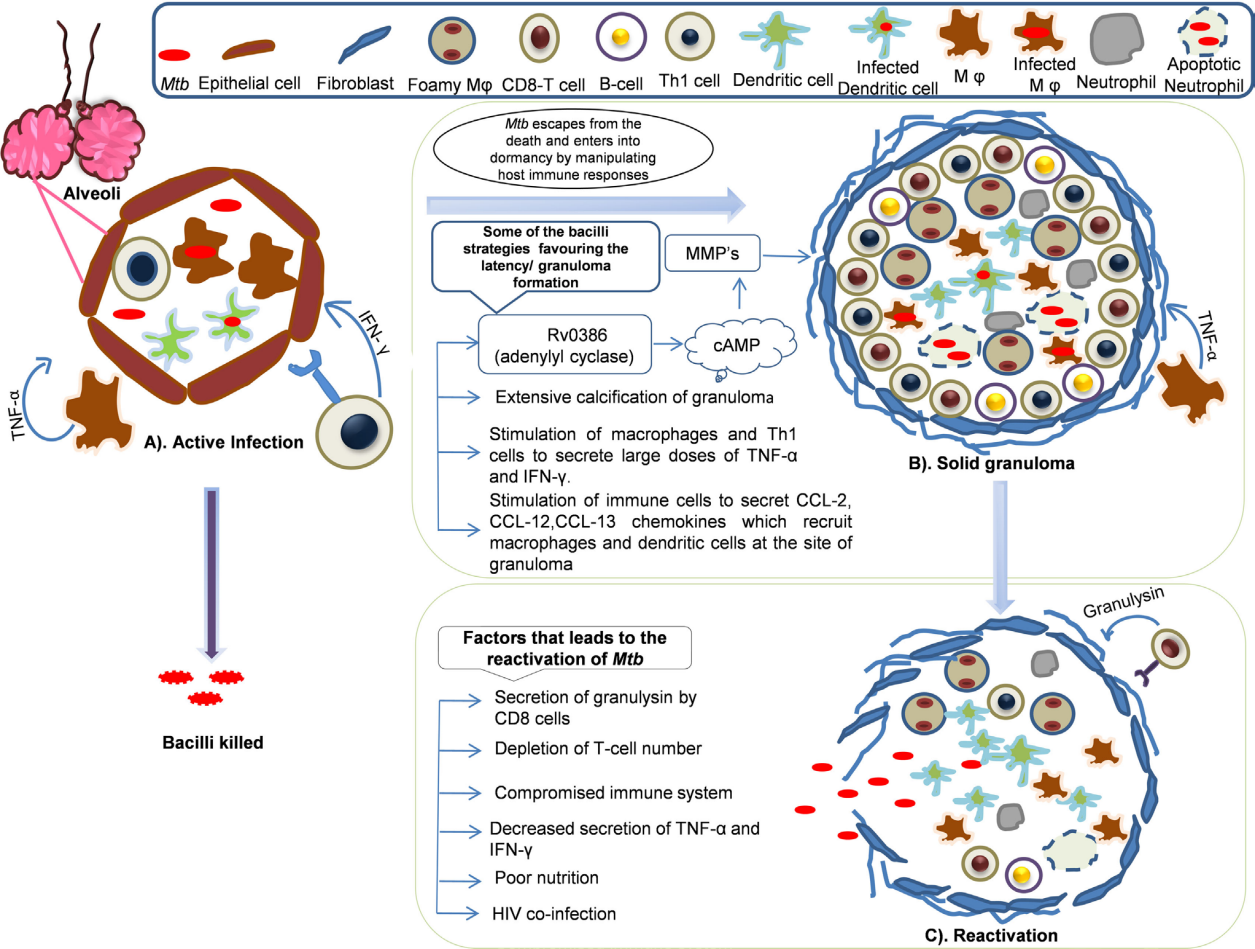
**Dynamics of granuloma formation, maintenance, and reactivation: *Mycobacterium tuberculosis* primarily harbors lungs and infects alveolar macrophages and establishes its niche**. The host defense sets in to counteract the actions *Mtb*. In this process, multiple possibilities exist where there could be **(A)** active infection/clearance: macrophages (Mφ) and Th1 cells secrete tumor necrosis factor (TNF)-α, IFN-γ that recruits other immune cells like neutrophils, dendritic cells (DCs), and B-cells that might clear the infection or bacilli may multiply leading to primary infection. However, some of the bacilli might escape the host’s immune actions and enter into dormancy. **(B)** Solid granuloma: solid granuloma is composed of macrophages, lymphocytes (B-cells and T-cells), DCs, and neutrophils. The solid granuloma is usually encircled by fibroblasts. During latent infection, *Mtb* encourages the immune system to form granuloma by manipulating host immune responses for its survival. Some of the *Mtb* survival strategies include stimulation of macrophages and T-cells to secrete large doses of TNF-α, chemokines such as CCL-2, CCL-12, and CCL-13, which are crutial for the recruitment of other immune cells and maintenance of granuloma. Extensive calcification of granuloma by *Mtb* leads to prevention of apoptosis. Secretion of *Mtb* antigens such as Rv0386 (adenylyl cyclase) produce cAMP which signal the synthesis of matrix metalloproteinases (MMPs) that are involved in the maintenance of granuloma by unknown mechanism. **(C)** Reactivation: *Mtb* reactivates and exits from the granuloma when immune system compromises and bacilli spread to new sites of infection. Poor nutrition, decrease in the number of T-cells, HIV coinfection are some of the contributing factor for reactivation.

In the granuloma lesions, either the infection is cleared or viability is promoted by creating a favorable niche to the bacterium (Figure 2) (115). The immune status of the human host and transcriptional signature of the bacteria directs early granuloma establishment and consequence of the disease (116). However, it is shown that the immunological responses to *Mtb* by infected individuals vary to a great extent and is dependent on the granulomatous lesion formed. Two kinds of granulomatous lesions were demonstrated in cynomolgus macaques that were infected with low doses of *Mtb* (117). The classical ones are the caseous granulomas with low oxygen availability that is characterized by fibroblasts surrounding the epithelial macrophages and neutrophils in the periphery and dead macrophages in the center (118), whereas fibrotic lesions are associated with latent TB and exclusively filled with fibroblasts with sparsely distributed macrophages (119). In the event of an immunocompromised condition, granulomas liquefy and the bacteria are released from degraded granuloma to re-infect lung tissue and spread to new hosts (120).

Although granuloma formation is a basic immune response elicited by the host against an infection, it is also promoted by *Mtb* as part of its virulence program (121, 122). TNF-α plays an important role in mounting a response that is cytotoxic to the pathogen as well as maintaining the structural integrity of the granuloma and this is evident by the fact that neutralization of TNF-α leads to fatal reactivation of the bacterium and increased burden of the bacilli in the lung tissues (123, 124). It is demonstrated that the lipid-rich mycobacterial cell wall composed of trehalose dimycolate induces TNF-α induction and thus the granulomatous inflammatory response (120). Eliciting an excessive TNF-α response is another mechanism of virulence of *Mtb* to favor its existence in the host without being detected. This is accomplished by increasing the levels of TNF-α in the macrophages by directly interfering with cAMP mediated responses. In the mutant strain of *Mtb* lacking *Rv0386* (encodes an adenylate cyclase), reduced protein kinase A and cAMP response element-binding protein activation was observed, which results in a significant reduction of macrophage TNF-α secretion (125). Although excess production of TNF-α is required for the containment of *Mtb*, this pathogen, however, uses adenylate cyclase to deliver excess cAMP to macrophage cytoplasm and acts to subvert host cell signal transduction so as to result in a pro-granulomatous response with excess TNF-α secretion (125).

The mechanisms of granuloma formation are not well studied. However, evidence points to the role of matrix metalloproteinases (MMPs) and tissue inhibitors of metalloproteinases (TIMPs) in *Mtb* for induction of granuloma formation (126). The expression of MMPs is largely dependent on cAMP-mediated signaling in the host cell (127) and thus the levels of these enzymes can be modulated by bacterial mechanisms (for example, the *Mtb Rv0386* pathway) that regulate cAMP levels. Secretion of MMP-1, -2, -7, and -9 and decreased expression of TIMP-1, -2, and -3 (126, 128) have been shown in the peripheral blood mononuclear cells and human airway epithelial cells. Governing granuloma formation is also exploited by this bacterium to increase its numbers and dissemination systemically. In the *Mycobacterium marinum*-zebrafish model, it was demonstrated that the infected macrophages attract new cells and they in turn ingest the bacterium and allow them to sustain, thus allowing the granuloma to expand (114). Thus, it is clear that *Mtb* pathogenesis involves subversion of host signaling in a pro-inflammatory manner to create an environment that is favorable for its dormancy.

### Regulation of Granuloma Maintenance

#### Calcification

Neutrophils on the other hand also display antimycobacterial activity and the mechanism involves generation of ROS (129, 130). Further, infected neutrophils undergo apoptosis (131, 132), thus contributing to an effective adaptive immunity during *Mtb* infection (133). However, *Mtb* adopts strategies to inhibit apoptosis and promotes necrosis of these cells by various mechanisms by expressing the region of difference 1 (RD1) and a type VII secretion system (ESX) (134). Apoptotic neutrophils are also subjected to necrosis because of the activation of Calpain, a Ca^2+^-activated protease (135) due to Ca^2+^ influx, and this process is generally termed as Ca^2+^-induced necrosis. The Ca^2+^ influx and the subsequent necrosis of neutrophils are promoted by ESAT, a mycobacterial leukocidin (136). Further, the dormancy regulator of DosR activates the Ca^2+^ ATPase of the plasma membrane of the microvesicles of mycobacteria to create hypoxic conditions (137). Thus, the calcification of neutrophils is an important survival strategy adopted by *Mtb* at the cellular level. On the other hand, it was reported that feeding *Mycobacterium paratuberculosis*-infected rats with low calcium enhanced their ability to clear the infection (138). Besides utilizing Ca^2+^ for its survival at the cellular level, *Mtb* infection causes extensive calcification in various host tissues. Calcification is observed in myocardial (139), pulmonary (140), musculoskeletal, central nervous, abdominal (141) and genitourinary systems (142), and in lymphatic tissue and more importantly the granulomas. During the granuloma formation, the parenchymal tissues calcify to form caseous structures, especially during chronic or latent infection (143). Calcification allows the formation of various granuloma structures within a single host, thus creating different microenvironments that are unique among them to allow the survival of *Mtb* (143). The molecular mechanisms of granuloma calcification remain unclear. However, calcification of granulomas allows a safe environment for *Mtb* to remain dormant for extended periods of time.

#### Fibroblasts

Besides macrophages, granulomas also contain other cells such as eosinophils, neutrophils, and fibroblasts. In the mature granulomas, fibroblasts are found in the periphery and embedded within collagen fibers. TB inflammation is associated with increased number of fibroblasts and the fibroblastic activity (144). These cells produce collagen bundles such that the fibrotic capsule separates the bacteria from the surrounding tissue. This process is a consequence of intra-granulomatous cytokine secretion (145). On the other hand, fibroblasts also participate in the burning out of granuloma (in the event of the pathogen completely eliminated) by secreting tissue inhibitors of metalloproteases (145). The dynamics of granuloma formation depends on the number and degree of differentiation of fibroblasts (144). Interferon-γ primed fibroblasts present bacterial antigens to Th1 cells for further processing. However, it was demonstrated that *Mtb*-infected fibroblasts fail to present the antigens (146), thus enabling these bacilli to evade immune response of Th1 cells. *Mtb* also directly affects the collagen turnover and expression of matrix metalloproteases and tissue inhibitors of matrix metalloproteases in fibroblasts (147). Thus, the manipulation of fibroblast function is one of the key strategies of *Mtb* for the establishment and sustenance of persistent infection.

### Genetics of Host and Pathogen in Mycobacterial Dormancy: A Perfect Storm?

The pathogenesis of *Mtb*, especially its ability to remain in dormant state and reactivation, depends on both the genetics of the host and the pathogen (148). This is evidenced by the fact that (a) dormant mycobacteria do not replicate at all and their cell divisions occur at a very low rate (149, 150) and (b) a high level of genomic stability was observed in these bacterium isolated from human populations. Hence, studies on the genetic variations of the *Mtb* and the host have invited a lot of interest in the past few years and the same are discussed in this section.

Host gene–environment interactions play a crucial role in determining the outcome of TB and these are very important to evolve strategies to prevent mycobacterial infection at the genomic level. Interestingly, the ability of the host genetic control depends on the mycobacterial strain encountered and the exposure intensity (151). Two major loci on chromosomal regions, 18q11.2 and 11p13, were found to be associated with incidence of TB (152, 153). TST reactivity and interferon-γ release assay responses during *Mtb* exposure were found to be hereditarily controlled (154, 155). Since heritability is related to latent tuberculosis infection (LTBI), the phenotype of certain genes seems to govern mycobacterial infection and the associated dormancy. For example, in a Ghanaian population, IL-10 promoter haplotype (−2849G/−1082G/−819C/−592C) in TST non-responders was significantly more compared to TST responders (15.3 vs. 9.7%, OR = 2.09, *p* = 0.01) (156). In individuals carrying GG genotype at 1082A>G, the prevalence of TST non-response was 1.5 times than those carrying the AA and AG genotypes (156). Further, SNPs in IL4 (−590T>C, *p* = 0.007) and IFN-γ (+874A>T, *p* = 0.02) genes are associated with TST response (156). A linkage of persistent TST negativity with chromosomal regions 2q21-2q24, 5p13-5q22 (157), 11p14 (also called TST1 locus), and 5p15 (also called TST2 locus) was identified (158). Thus, the genetics of the host determines the susceptibility to mycobacterial infection and the development of LTBI.

Dormancy in *Mtb* is regulated by a set of approximately 50 genes, the DosR regulon, under the tight control of the dormancy survival regulator transcription factor (159). The genes of the DosR regulon are distributed in nine blocks in the genome: (block 1) *Rv0079–Rv0081*, (block 2) *Rv0569–Rv0574c*, (block 3) *Rv1733c–Rv1738*, (block 4) *Rv1812c–Rv1813c*, (block 5) *Rv1996–Rv1998c*, (block 6) *Rv2003c–Rv2007c*, (block 7) *Rv2028c–Rv2032*, (block 8) *Rv2623–Rv2631*, and (block 9) *Rv3126c–Rv3134c* (160). Evolutionarily, these genes are conserved across various pathogenic, non-pathogenic, and environmental bacteria of diverse habitats (160), and this could have occurred due to horizontal gene transfer mechanism during adaptation to challenging environmental conditions (161). Functionally, they emerged primarily to assist *Mtb* to adapt for anaerobic environment, thus enabling its survival in the host granuloma (162). Their expression is induced under hypoxia (163, 164) and under conditions where mycobacterial growth is inhibited by external growth factors both *in vitro* (macrophages) (165) and *in vivo* (mice and guinea pigs) (166, 167), suggesting their role in maintaining a low profile of bacterial growth under unfavorable conditions. Interestingly, the protein products of many of these genes seem to be good T-cell antigens and involved in many physiological processes of both the bacilli and the host.

The protein products encoded by DosR genes are predicted/reported to be involved in various functions, and on this basis, they were classified into eight groups (160). The functional roles of these genes are outlined in Table 1. Although they were predicted to have diverse functions, immune regulation and modulation of host responses have gained importance in the recent years. Out of the DosR genes expressed during dormancy, 18 are T cell responders that trigger strong IFN-γ response in TB patients. Further, it was demonstrated that *Rv1733c, Rv2029c, Rv2627c*, and *Rv2628* are strong IFN-γ responders in latently infected individuals (168). Further, *Rv2032, Rv1998c, Rv2031c, Rv2623*, and *Rv3132c* exhibit strong T-cell response, whereas *Rv0079, Rv0080, Rv3127, Rv2626c*, and *Rv2029c* exhibit strong humoral immune response (169, 170). It was identified that *Rv2626c* is a secretory protein, which binds to macrophage affecting its function and also elicit TNF-α and strong B-cell responses (171). In aerosol-mediated murine TB model, decreased induction of pro-inflammatory cytokines (IL-1 and IL-12) and decreased bacterial load and delayed death was observed when infected with *Rv1813c* deletion mutant of *Mtb* (172). It is to be noted that evidence for the role of DosR genes in *Mtb* dormancy stems out from murine models and no concrete evidence exists in higher animal models. In a recent study, using macaque as the model system, it was demonstrated that during hypoxic conditions *DosR* regulon modulates the timing and magnitude of adaptive immune responses to favor persistence of infection by *Mtb* (173).

**Table 1:** **Functional role of proteins encoded by the DosR genes**.

**Predicted functional group**	**Gene**	**Reported/predicted functional role of the encoded protein**
Stress response	Rv2623	Regulates bacillary growth of *Mtb* by modulating ATP-binding (174). Biomarker for the diagnosis of latent tuberculosis meningitis (175)
	Rv3134c	Associates with devRS to form an operon that mediates the expression of DevR target genes (176)
	Rv2031c	Alpha-crystallin (Hsp-X), a master regulator of Rv-2018-2031 operon (177); blocks the differentiation of monocytes to dendritic cells (178)
	Rv2624c, Rv1996, Rv2005c, Rv2028c	Not yet characterized

Proteases and transport	Rv2625c	Metalloprotease (Rip3); required for early phases of pulmonary tuberculosis infection (179)
	Rv1997	Not yet characterized
	Rv1735c	Immunogenic (169)
	Rv1733c	Enhances humoral and cellular immunity (180)

Host–pathogen interactions	Rv2626c	Modulates macrophage effector functions and delayed hypersensitivity (171). Diagnostic marker for tuberculosis (181)
	Rv2004c	Binds specifically to U397 macrophages and A549 epithelial cells to modulate immune responses (182)

Sensor kinases and transcriptional regulation	Rv3132c (DosS), Rv2027c (DosT)Rv3133c (DosR)	Form a two sensor kinase system (183)
	Rv0081	Predicted to involve in encoding of formate Hydrogenylase complex (Rv0081-Rv0088 locus) (184); highly immunogenic in African populations (169)

Cell wall and protein synthesis	Rv0079	*RafH* (ribosome-associated factor during hypoxia); stabilizes ribosome under stress conditions (6, 185); inducer of T-cell responses (186)
	Rv0574c	A pyroglutamate synthase-like protein, modulates poly-α-l-glutamine content in the cell walls to maintain cell integrity under hostile conditions (187)
	Rv1738	Shutdown of ribosomal protein synthesis (188)

Nucleotide metabolism and repair	Rv2630	Immunogenic in patients with active pulmonary tuberculosis; exact role in nucleotide metabolism and repair are yet to be proven *in vivo* (189)
	Rv2631	RNA-splicing ligase RtcB; function not yet characterized
	Rv0570	Putative vitamin B12 dependent ribonucleoside-diphosphate reductase; immunogenic (134)
	Rv0571c	Putative phosphoribosyltransferase; function not yet characterized

Nitrogen metabolism	Rv3131	Putative NAD(P)H nitroreductase and immunogenic in nature (169, 190)
	Rv2032	Putative NADP(H) nitroreductase (191); Potent inducer of host cytokines (192, 193)
	Rv1737c, Rv1736c	Nark2 and NarkX (encoded by Rv1737c and Rv1736c, respectively) are nitrate/nitrite transporters required during mycobacterial anerobic dormancy (194); immunogenic and potent diagnostic markers of tuberculosis (169)
	Rv3127	Not yet characterized

Redox balance	Rv0573c	Predicted to involve in biosynthesis and recycling of nicotinamide; lmmunogenic (195)
	Rv1812c	Nitrogen metabolism during stress (172)
	Rv3130c	Putative Diacylglycerol *O*-acyltransferase; facilitates accumulation of triacylglycerol under stress (196)
	Rv2029c	Putative 6-Phosphofructokinase (PfkB); induces cytokine production (197)
	Rv1734c, Rv2006, Rv1998c, Rv2003c, Rv2007c	Not yet characterized

Hypothetical proteins	Rv2628	Immune-mediated protection against tuberculosis (198)
	Rv2627c	Delays mycobacterial growth (199)
	Rv2629, Rv3126c, Rv0569, Rv0572c, Rv0080, Rv2030c, Rv3128c, Rv1813c	Not yet characterized

It is very interesting to note that mutations in dosR does not induce *Mtb* death under hypoxic conditions, indicating that other factors beyond dosR are important for the dormancy and survival of *Mtb* in the host. Further, the massive expression of dosR-independent genes during hypoxic conditions and the variation in the expression profiles of dosR genes in different strains of mycobacteria of different virulence indicates that further studies are required to warrant the exclusive role of these genes in mycobacterial dormancy (200–202).

### Environmental Signals of Sleep

The complex life cycle of *Mtb* involves adaptation to various stresses and to accomplish this it encodes about 190 regulatory proteins among which 11 form the two-component signal transduction systems (TCSSs) (134, 203). TCSSs found in *Mtb* are conserved in other closely related mycobacterial species in terms of genetic arrangement and location (134, 204). However, the number of functional TCSSs seems to vary between the species, wherein the *Mycobacterium leprae* had only four TCSSs (204). The TCSSs identified in *Mtb* are phoP-phoR, regX3-senX3, dosR-dosS (dosT), Rv0600c-Rv0601-ctcrA, narL-Rv0845, tcrX-tcrY, mprA-mprB, prrA-prrB, trcR-trcS, pdtaR-pdtaS, mtrA-mtrB, and kdpD-kdpE (205).

A typical TCSS comprises a histidine sensor kinase and a response regulator that are localized in the plasma membrane and cytoplasm, respectively. Both of them have specific domains through which they sense environmental cues. The sensor kinase comprises a sensor domain, one or more transmembrane domains, and a cytoplasmic transmitter containing a dimerization motif and a kinase domain, and the latter can be again divided into two subdomains possessing a histidine phosphorylation box and an ATP-binding pocket (made of N, D, F, and G boxes that have highly conserved amino acids); signal recognition results in dimerization and auto-phosphorylation followed by the transfer of this phosphate to the response regulator, thus enabling it to promote transcriptional, translational, and functional aspects (206–209). The environmental cues that activate the TCSSs are not yet defined for all of them. Inorganic phosphate (regX3-senX3); SDS, triton X-100 alkaline pH, and nutrient limitation (mprA-mprB); low oxygen, nitric oxide, carbon monoxide, and ascorbate (DosS-DosR) are some of the signals that are identified to activate the TCSSs in model organisms such as *M. smegmatis*. However, whether the same signals also activate these TCSSs in *Mtb* is not yet clear. The signaling mechanisms of some of the characterized TCSSs remain more or less similar as described above for a typical TCSS, with some variations. One of the downstream events of TCSS activation is gene regulation. SenX3-RegX3 activation leads to the upregulation of several genetic determinants such as phoA (alkaline phosphatase) (210), *pstSCAB* and *phnDCE* (encodes phosphate-specific transporter systems) (210, 211), and phnF (encodes a negative regulator of phnDCE) (211) and SenX3-RegX3 itself (210, 212). PhoP-PhoR regulates about 150 genes that are involved in general and lipid metabolism, and respiration (213, 214). Further, a number of genes that code for membrane proteins, genes of dosRS regulon, genes of the PE/PPE/PE-PGRS protein families, and genes of the virulence-associated RD1 such as espB and espR (214, 215). The narL-Rv0845 TCSS regulates genes involved in nitrate metabolism during anaerobic respiration (216). mprA-mprB differentially regulates about 200 genes (217, 218). Examples include expression of its own gene, pepD and moaB2 and Acr2 (alpha-crystallin-like protein) (219, 220). The trcR-trcS TCSS regulates about 50 genes (221). dosR-dosS (dosT), one of the well-characterized TCSS, regulates about 48 genes (collectively called the DosR regulon), predominantly having a role in hypoxia. MtrA-MtrB seems to be an important TCSS since it regulates genes involved in DNA replication and cell wall integrity (222, 223). The gene expression regulated by pdtaR binding to RNA involves prevention of stem-loop structure formation by acting as an anti-terminator (224). The huge variety of genes regulated by TCSSs gives an edge for *Mtb* to create favorable conditions for its survival in the host in a dormant condition for longer durations.

Evidence to strengthen the crucial role of TCSS is demonstrated in animal models of TB infected with mycobacterial strains that harbor mutation in the TCSS genes. Attenuation of bacterial growth in lung, liver, and spleen and delayed time to death of *Mtb*-infected animals are some of the phenotypes described in studies that used TCSS mutants [reviewed in Ref. (205)].

## Reactivation

### Reactivation and Liquefaction of the Granuloma

**Figure 3:**
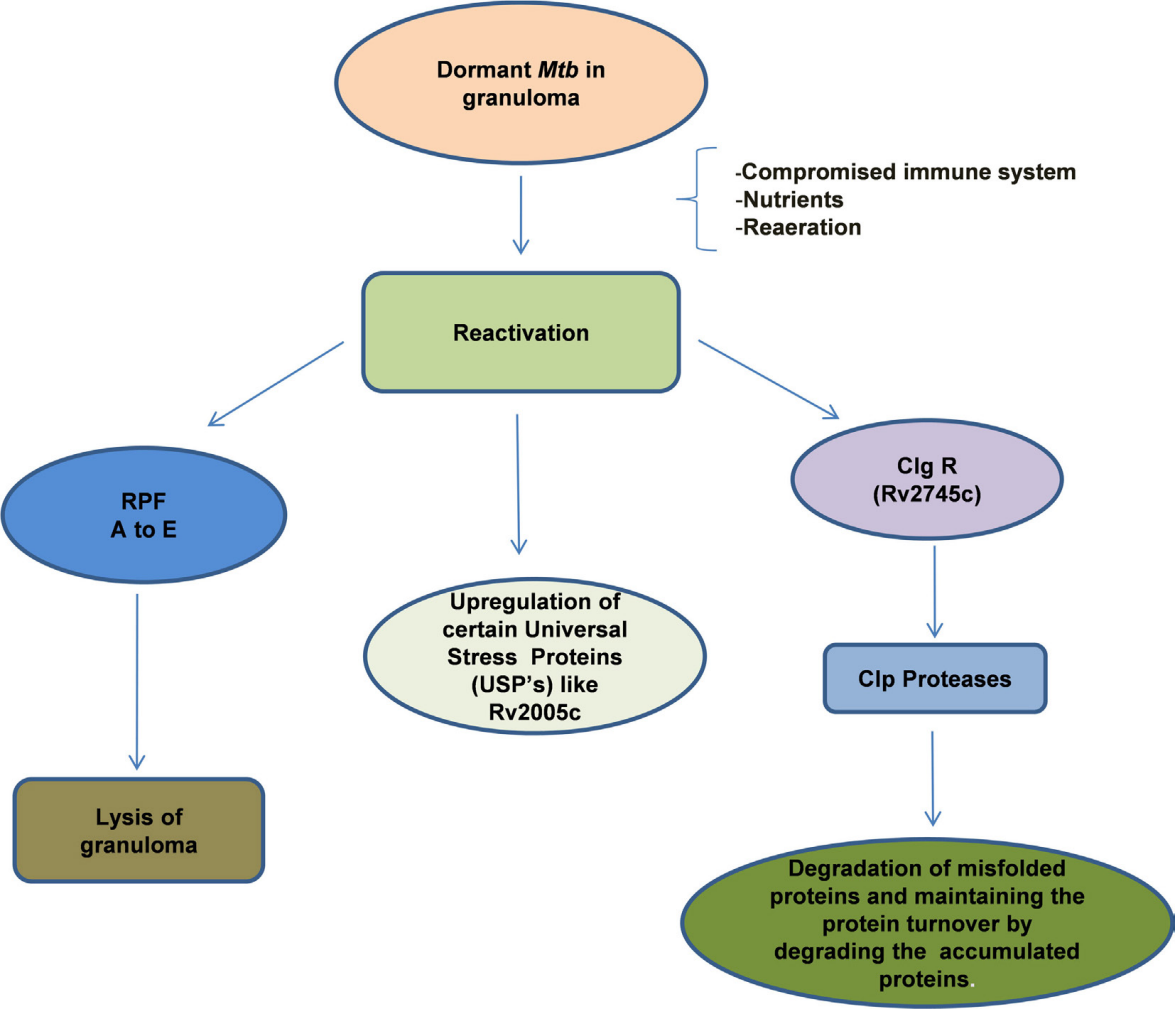
**Reactivation of latent bacilli: *Mycobacterium tuberculosis* (*Mtb*) persisting under hostile conditions in granuloma reactivates when favorable conditions prevail such as availability of nutrients, re-aeration, and immune compromization of the host leading to activation of different set of genes that are required for the re-growth of the bacilli**. During reactivation, *Mtb* mainly expresses Clp protease gene Regulator (ClpgR) and its inducible genes Clp Proteases. Clp proteases play a vital role in the maintenance of protein turnover, degradation of misfolded or accumulated proteins, helping the bacteria to shift to metabolic phase. On the other hand, *Mtb* expresses a family of five resuscitation-promoting factors (RPF A to E) that are essential for the lysis of granulomatous cell wall. Certain Universal stress proteins (like Rv2005c) are also upregulated during resuscitation which might play an essential role for the re-growth.

The reactivation of dormant *Mtb* is governed by a group of proteins belonging to the resuscitation-promoting factor (RPF) family and their genes were found to be upregulated during this process (149, 225, 226) (Figure 3). In general, these genes are upregulated when the stress is removed. The importance of these genes was demonstrated by the fact that *Mtb* in which *Rpf* genes were knocked down, were unable to undergo reactivation even after immune suppression of the host (227, 228). The five Rpf genes (A to E), though, not required for general viability, are crucial for the induction of reactivation of *Mtb* and the bacilli can survive multiple mutations across the underlying genes. The Rpf proteins conserve a domain that is structurally close to lytic transglycosylases and are thought to participate in cell wall hydrolysis, an essential early phase step in the reactivation or resuscitation process (229). The action of these proteins breaks the peptidoglycan strands of the highly impermeable cell wall of the granulomatous cells. During the reactivation process, increase in cAMP levels due to activation of adenylate cyclase by free fatty acids is evident and the RPF proteins do not seem to be involved in this process (230). However, gene expression analyses indicated that the RPF biosynthesis is active in the later phase of reactivation, suggesting that they may mediate the early and late events (230, 231). Thus, each of these genes is considered as a potential drug target that would allow *Mtb* to exit the dormant stage for further treatment by conventional drugs.

Besides the Rpf genes, the DosR regulon gene (Rv3133c) also contributes to the rapid resumption of *Mtb* growth especially when they transit from the non-respiring conditions to the respiring conditions (200). The dosR regulon also regulates the expression of non-coding short RNAs that are involved in both dormancy and reactivation (232). A recent study indicates that Clp protease gene regulator, Rv2745c (clgR) is required for *in vitro* reactivation from hypoxia-induced dormancy (233). In the isogenic mutant *Mtb*:ΔRv2745c, genes in the σ(H)/σ(E) regulon as well as the dosR regulon were dysregulated suggesting that the DosR genes are regulated at a different level to contribute to the reactivation process (233). It thus appears that *Mtb* reactivation involves biochemical, immunological, and genetic aspects all of which are potential drug targets for the development of treatment strategies to prevent dormancy and reactivation of the bacilli.

### Exiting the Granuloma: In Search of a New Niche

Besides the biochemical aspects that favor apoptosis or necrosis, genetic reprograming also plays a vital role in the reactivation of *Mtb* from its inactive state (234). The prime necessity of any pathogen to enter a dormant phase is to establish its niche and re-infect when the conditions are favorable. Evolutionarily, the pathogens exit the host in which they have resided to infect a new host. In the case of *Mtb*, the bacilli exit from the granulomas that have undergone necrosis in to the bronchial tree and this seems to be most efficient method of transmission to the new host (235, 236). Reactivation generally occurs when the host immune machinery is weakened or suppressed due to physiological or pathological factors. For example, in the case of HIV^+^ individuals, increased risk of *Mtb* reactivation is possible due to the low levels of CD4^+^ T cells (237). Further, the immune responses during reactivation differ from that of primary infection with CD8^+^ T cells taking the lead than the CD4^+^ T cells (238). In a primate model exhibiting latent TB and HIV coinfection, it was observed that animals with low CD4^+^ T cells show higher incidence of TB suggesting that T-cell depletion is one of the major triggering factor for *Mtb* reactivation (239). Though these observations were confirmed in smaller animal models, controversy still exists in the primates and humans (240).

Further the immunological factors, cellular events also determine the efficiency of reactivation. The nature of cell death experienced by the granuloma cells defines the extent of infection (111). Apoptosis of the macrophages allows the bacilli to remain encased in the macrophages, which then are phagocytosed by new macrophages and thus allowing bacterial expansion or maintenance in the new granuloma cells. On the other hand, necrosis of macrophages in the granuloma releases the bacilli into the extracellular milieu allowing multiplication in high numbers and these new bacilli structurally appear as serpentine cords. It is very interesting to note that the serpentine cords are not readily recognized by macrophages, thus allowing them to successfully get transmitted to a new individual (241). What factors and conditions that allows a granuloma to undergo apoptosis or necrosis is still a matter of debate and is being actively investigated. Once in the airway mileu, the new bacterial cells are aerosolized in cough droplets.

### Anti-Cytokine Inhibitors: Contribution to Reactivation

As discussed earlier, TNF is a potent inflammatory cytokine that controls the dynamics of pathogen survival and host immune response. Among the cytokines implicated in *Mtb* pathogenesis, TNF is a potent inflammatory cytokine that controls the dynamics of pathogen survival and host immune response. TNF confers anti-TB immunity to the host by manipulating the levels of other cytokines, adhesion molecules, and apoptosis of macrophages. In the clinical settings, TNF blockers such as infliximab, adalimumab, certolizumab pegol, and etanercept are routinely used for the treatment of various autoimmune disorders. An emerging concern is the association between the use of TNF inhibitors and increased risk of *Mtb* reactivation. It is observed that treatment with TNF inhibitors resulted in progression of *Mtb* from latent to reactivation (242, 243). Recent studies project a higher risk of TB in rheumatoid arthritis patients receiving TNF inhibitor treatment (244). Further, in various animal models, it is demonstrated that neutralization of TNF resulted in increased susceptibility to primary infection of *Mtb* (245, 246). TNF inhibitors interfere with innate and adaptive immune responses such as increased T-cell activity, complement-mediated lysis, apoptosis of immune cells, and phagosomal maturation (247). The changes that occur in the immune responses due to TNF inhibitors give an opportunity for *Mtb* to reactivate. A thorough multistep screening for *Mtb* is proposed for individuals who are subjected to anti-TNF therapy to treat autoimmune diseases (248).

### Clinical Implications of Dormancy/Therapeutic Manipulations of Dormancy

The unique feature of *Mtb* is its ability to maintain a persistent infection without being detected under different conditions has a lot of impact on the clinical implications. *Mtb*, because of its ability to create a secure environment for itself is not susceptible to certain antibiotics and also resistant to strong antibiotics such as isoniazid (249). Development of drug resistance during dormancy is mainly due to chromosomal mutations in genes required for antibiotic action. Isoniazid (INH) and rifampicin (RIF) are the front line drugs for the treatment of TB. However, over a period of time, *Mtb* developed multidrug resistance and currently a combination of 8–10 drugs are being used for treating MDR-TB. Development of multidrug resistance by *Mtb* complicates the clinical interventions. Serious side effects such as nephrotoxicity, ototoxicity, and dysglycaemia due to the use of powerful anti-TB drugs such as aminoglycosides, ethionamide, and gatifloxacin are some of the indirect clinical implications caused by the ability of *Mtb* to acquire drug resistance (250). Another clinical implication that is very serious is the re-emergence of TB when host immune responses fail in conditions such as HIV infection (237) and the increased risk of developing TB in patients treated with anti-TNF (251). Further, corticosteroid therapy, deficiency of vitamin D, and other possible conditions that affect T cell function increases the risk of TB (252). The main therapeutic implication of mycobacterial dormancy is development of drug resistance and this is mainly due to chromosomal mutations in genes required for antibiotic action.

## Conclusion

Despite global efforts to tackle incidence and transmission of TB, about 10 million people are diagnosed with this disease each year, leading to approximately 2 million deaths. A minority of the infected patients enjoy total elimination of the pathogen but, a majority of the cases appear to contain *Mtb* in dormant phase. Identification and diagnosis of individuals with latent TB has been an active area of investigation even in the era of advanced molecular and cell biology. Although the role of many genes and their protein products that contribute to dormancy and reactivation of *Mtb* were studied and proposed as potential targets/antigens for the development of drugs and vaccines, the application of the same at the ground level in containing TB still remains a major challenge. Whether or not the functional applicability identified for these genes is sufficient to develop control strategies, such as development of vaccines against TB will need to be tested, evaluated, and addressed. If so, would the vaccines be designed with multiple antigens encoded by the genes involved in dormancy and reactivation of *Mtb*? On the same lines, should multiple antigen strategy be adopted in designing highly sensitive diagnostics that can detect stage-specific antigens of *Mtb* to allow the option of a strategic treatment protocol and prevent the “under representation” of dormant *Mtb* cases as healthy individuals? On the other hand, can the immune system of the host be manipulated to efficiently prevent dormancy of *Mtb*? What is the relevance of the functional aspects of genes involved in latency and reactivation for the development of extreme drug resistance in *Mtb*? Answering all these questions, although difficult form a practical point of view, would enable us tackle *Mtb* by a multipronged approach that involves prevention, timely detection of infection, and also the identification of the stage of infection through the development of novel drugs that can target the pathogen at all the three stages, namely, infection, dormancy, and reactivation.

## Author Contributions

VP: collected the data and developed the entire manuscript. SD: sketched the figures and participated in discussions for developing the ideas centered on dormancy regulon gene functions. NA: provided overall concept and thought leadership and edited the final version of the manuscript. All the authors have read and approved the final manuscript.

## Conflict of Interest Statement

The authors declare that the research was conducted in the absence of any commercial or financial relationships that could be construed as a potential conflict of interest.
